# Editorial: Resistant pathogens: from distribution to emerging therapies

**DOI:** 10.3389/fmicb.2023.1243067

**Published:** 2023-07-04

**Authors:** Estéfani García-Ríos, Michael J. McConnell, Pilar Pérez-Romero, Mireia López-Siles

**Affiliations:** ^1^Infecciones Víricas e Inmunidad en Enfermos Inmunodeprimidos, National Centre for Microbiology, Instituto de Salud Carlos III (ISCIII), Madrid, Spain; ^2^Reference and Research Laboratory in Resistance to Antibiotics and Infections Related to Healthcare, Intrahospital Infections Unit, National Centre for Microbiology, Instituto de Salud Carlos III (ISCIII), Madrid, Spain; ^3^Microbiology of Intestinal Disease, Biology Department, Universitat de Girona, Girona, Spain

**Keywords:** antibiotic resistance, antifungal resistance, resistome, vaccine, Extended-spectrum ß-lactamase (ESBL), *Staphylococcus aureus*, *Staphylococcus pseudintermedius*

The emergence and spread of drug resistance is a public health concern problem globally. This phenomenon includes resistance to antibiotics but also other antimicrobials such as antivirals, antifungals, and antiprotozoals.

According to the World Health Organization, antibiotic resistance is currently one of the greatest threats to global health, food security, and development. It has been estimated that if current trends continue, by 2050, 10 million deaths will occur due to diseases caused by antibiotic-resistant microorganisms (O'Neill, 2014). In this sense, a prioritized list of pathogens of critical concern for which the development of new antimicrobials is encouraged has been published (Tacconelli et al., 2018). Resistant strains of Gram-negative bacteria of species such as *Acinetobacter baumannii, Pseudomonas aeruginosa*, and certain Enterobacteriaceae species are on top of the list. In addition, strains of Gram-positive bacteria are among pathogens of high priority and include resistant *Enterococcus faecium* and *Staphylococcus aureus*, among others.

On the other hand, fungal infections affect more than 300 million people globally, resulting in 1,660,000 deaths in developing countries (Almeida et al., 2019). In fact, 90% of all deaths from fungal infections are due to fungi of the genera *Cryptococcus, Candida, Pneumocystis*, and *Aspergillus* (WHO, 2022), and antifungal resistance is also an emerging and worrying issue in the field of medical mycology.

The number of therapeutic options currently available against these pathogens is limited, partly due to the rapid appearance and spread of resistance. In addition, the development of new drugs for treating these infections with the approaches used to date will likely not be sufficient for confronting antimicrobial resistance at the global level. Therefore, conventional strategies currently used to develop new antimicrobials have to be reconsidered and novel avenues should be explored.

This Research Topic collects articles that aim to gain an increased understanding of the distribution, mechanisms of resistance, and potential novel strategies that are being explored to fight resistant microorganisms.

Surveillance and epidemiology of resistant pathogens in both clinical and community settings are key to monitoring the spread of resistance and to developing public health policies. While adult populations have been the focus of attention in most studies, limited data exist regarding the distribution of resistant strains in children. An original research article by López-Siles et al. determined the prevalence of Extended-spectrum ß-lactamase-producing Enterobacterales (ESBL-E) in fecal samples of 887 healthy Spanish schoolchildren. ESBL-E prevalence was below 3% and *Escherichia coli* was the main ESBL-producing species detected. Although strains harbored ESBLs of different families, that of CTX-M was the most frequently identified ESBL. Polyclonal dissemination was the most likely mechanism of spread, as high ST diversity was observed among isolates. Of note, antimicrobial resistance profiles of isolates evidenced co-resistance with other antibiotics, and this was particularly frequent in ST131 strains.

The development of new therapies to treat resistant pathogens is of utmost importance for fighting these pathogens. Two original research articles published in this Research Topic reported novel methodologies. Yu et al. explored a therapeutic strategy against *S. aureus* infection by reducing alpha-hemolysin expression with a natural product, *i.e.*, 2,3-dehydrokievitone. This study demonstrated that the compound decreased *agrA* transcription levels. As AgrA is a global regulator of virulence, this strategy may target several virulence factors and toxins of the microorganisms, an approach that may not promote the selection of antibiotic-resistant strains. Of note, the compound was tested in an animal model of experimental pneumonia, and the mice receiving the compound had significantly reduced mortality and less severe pneumonia and bacterial burden. Jaan et al. explored a different approach based on subtractive proteomics and reverse vaccinology to identify targets for potential novel drugs and vaccines against *Staphylococcus pseudintermedius*. After screening the core-proteome of the species, over 30 novel potential drug targets were identified. In addition, two proteins were potential candidates for vaccine development and a chimeric multi-epitope vaccine was designed. Subsequent *in silico* human body immune response prediction confirmed immunoglobulin binding affinity. Therefore, this study significantly contributed to identifying novel therapeutic targets to fight this pathogen.

While bioinformatics approaches to predict bacterial resistance have been developed over the last decade, similar tools for antifungal resistance prediction are scarce. The perspective article by Alastruey-Izquierdo and Martín-Galiano underscores the main challenges that must still be overcome. These include the limited number of databases collecting data on mutations, leading to resistance in antifungal targets with subsequent laboratory validation, a better understanding of resistance mechanisms, the development of appropriate bioinformatics analysis pipelines, and the need for standardized protocols, among others. The worrisome epidemiological data and the limited number of antifungals underscore the need for controlling difficult-to-treat fungal pathogens, and in this sense, resistome predictors similar to those already implemented for bacteria are urgently needed.

In conclusion, understanding the spread of resistant pathogens and developing new therapeutic options is key to preventing and controlling infections by these challenging pathogens. In addition, improvements in diagnostic and prognostic methods may also help to better detect these pathogens, characterize their mechanisms of distribution, and predict the outcomes of infections. Understanding the mechanisms of antimicrobial resistance, virulence, and adaptation to stressful conditions is also important for preventing and controlling infections caused by these microorganisms. In addition to resistant bacteria and fungi, future insight should also consider viral and protozoa infections. Altogether, an integrated strategy is required to fight the health and economic challenges that infections by resistant microorganisms represent.

## Author contributions

All authors listed have made a substantial, direct, and intellectual contribution to the work and approved it for publication.
